# Association between chronic periodontitis and the risk of Alzheimer’s disease: a retrospective, population-based, matched-cohort study

**DOI:** 10.1186/s13195-017-0282-6

**Published:** 2017-08-08

**Authors:** Chang-Kai Chen, Yung-Tsan Wu, Yu-Chao Chang

**Affiliations:** 1 School of Dentistry, Chung Shan Medical University, No. 110, Section 1, Jianguo N. Road, Taichung City, 40201 Taiwan; 2 Section of Dentistry, Zuoying Branch of Kaohsiung Armed Forces General Hospital, Kaohsiung, Taiwan; 3 Department of Physical Medicine and Rehabilitation, Tri-Service General Hospital, School of Medicine, National Defence Medical Centre, Taipei, Taiwan; 4 Department of Dentistry, Chung Shan Medical University Hospital, Taichung, Taiwan

**Keywords:** Alzheimer’s disease, Periodontitis, Risk factors, Nationwide population, Cohort study

## Abstract

**Background:**

Although recent short-term cross-sectional studies have revealed that chronic periodontitis (CP) may be a risk factor for increased cognitive impairment in patients with Alzheimer’s disease (AD), systematic reviews and long-term longitudinal studies have provided less clear evidence regarding the relationship between CP and AD. Therefore, we conducted a retrospective cohort study using the National Health Insurance Research Database (NHIRD) of Taiwan to determine whether patients with CP are at increased risk of developing AD.

**Methods:**

We conducted a retrospective matched-cohort study using the NHIRD of Taiwan. We identified 9291 patients newly diagnosed with CP between 1997 and 2004. A total of 18,672 patients without CP were matched to the patient cohort according to sex, age, index year, co-morbidity and urbanisation level. Cox proportional hazards regression analyses were performed to evaluate the subsequent risk of AD.

**Results:**

Patients with CP had a higher prevalence of hyperlipidaemia, depression, traumatic brain injury and co-morbidities, as well as higher urbanisation levels, than those in the unexposed cohort (all *p* < 0.01). At the final follow-up, totals of 115 (1.24%) and 208 (1.11%) individuals in the CP exposed and unexposed groups, respectively, had developed AD. Patients with 10 years of CP exposure exhibited a higher risk of developing AD than unexposed groups (adjusted HR 1.707, 95% CI 1.152–2.528, *p* = 0.0077).

**Conclusions:**

Our findings demonstrate that 10-year CP exposure was associated with a 1.707-fold increase in the risk of developing AD. These findings highlight the need to prevent progression of periodontal disease and promote healthcare service at the national level.

## Background

Alzheimer’s disease (AD) is a neurodegenerative disease characterised by progressive cognitive decline and memory loss, inevitably leading to complete loss of mental faculties and death [1]. AD is the most common cause of dementia in older adults [2–5]. Furthermore, due to increasing life expectancy and lifestyle changes, recent projections have indicated that 1 in 85 individuals will be diagnosed with AD by 2050 [6].

Recent evidence indicates that peripheral infections, blood vessel damage and oxidative stress may aggravate inflammation in the brain and play an important role in the pathogenesis of dementia and AD [7, 8]. Indeed, previous reports have revealed that diabetes mellitus [9], hypertension [10], hyperlipidaemia [11], chronic kidney disease [12], depression [13], stroke [14], traumatic brain injury [15] and periodontal problems [16, 17] are associated with cognitive impairment. Chronic periodontitis (CP) is a peripheral infectious/inflammatory condition that is among the leading risk factors for tooth loss [18]. CP has been associated with increases in serum levels of C-reactive protein (CRP) [19] and pro-inflammatory cytokines (e.g., tumour necrosis factor-α), as well as decreases in anti-inflammatory markers (e.g., interleukin-10) [20]. Studies have further revealed that CP is linked to numerous inflammatory diseases, including cardiovascular disease [21] and diabetes mellitus [22], as well as to other neurodegenerative disorders, such as Parkinson’s disease [23]. Researchers have speculated that this association is due to the increases in systemic inflammation that accompany the growth of periodontal pathogenic microorganisms. In other mechanism by which CP contributes to AD, systemic inflammation caused by periodontal pathogens may also play a role in vascular disease and endothelial dysfunction. With accumulating studies supporting vascular factors in the development of AD, vascular factors could be a mediator in the development of AD [24–26].

Although recent short-term cross-sectional studies have revealed that CP may be a risk factor for increased cognitive impairment in patients with AD [5, 27], systematic reviews and long-term longitudinal studies have provided less clear evidence regarding the relationship between CP and AD [1, 26, 28]. Therefore, we conducted a retrospective cohort study using the National Health Insurance Research Database (NHIRD) of Taiwan to determine whether patients with CP are at increased risk of developing AD.

## Methods

### Data sources

Developed in 1995, the National Health Insurance Program provides universal and comprehensive healthcare for approximately 99% of residents in Taiwan [29]. In the present study, we used 1996–2013 data from the NHIRD in Taiwan. One million individuals included in the NHIRD were selected at random, representing about 4.5% of all enrolees [30]. There was no significant difference in age or sex between participants included in the study sample and all NHIRD enrolees. We extracted data regarding demographic characteristics, including encrypted identification numbers, sex, dates of birth and death, and diagnostic information. The diagnostic data included the dates of dental procedures, as well as International Classification of Diseases, Ninth Revision, Clinical Modification (ICD-9-CM), diagnostic and procedure codes [31]. Approval for the present study was obtained from the institutional review board (IRB) of Chung Shan Medical University (CS2-15071). The requirement for informed consent was waived by the IRB because all NHIRD data had been de-identified.

### Study design and sampled participants

The present retrospective, matched-cohort study included patients aged ≥50 years who had been newly diagnosed with CP (between 1 January 1997 and 31 December 2004) on the basis of ICD-9-CM diagnostic criteria: code 523.4 (CP) [32]. In addition, each enrolled patient had been diagnosed with CP following at least two outpatient clinic visits during the 1-year study period [33]. Exclusion criteria were as follows: (1) unknown age and/or sex, (2) diagnosis of CP prior to 1997 and (3) diagnosis of AD (ICD-9-CM code 331.0) prior to 1997 or prior to the first visit for CP [33]. To ensure the accuracy of our findings, we also excluded patients with unknown vital status and those who had not used health services in the past 12 months, because the NHIRD does not include death records. The sample included a total of 9291 patients with CP and 18,672 patients without CP matched according to sex, age and index years (1:2 ratio).

**Fig. 1:**
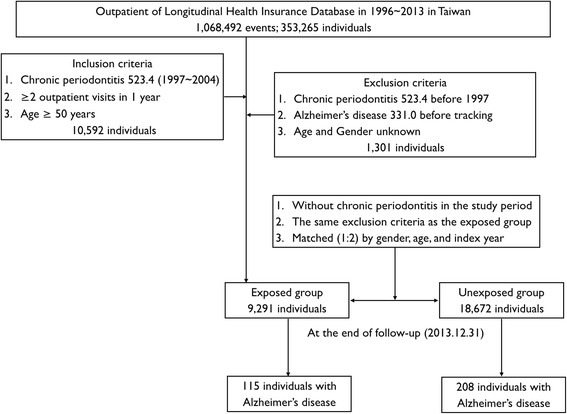
Flowchart of cohort selection of patients from the National Health Insurance Research Database in Taiwan

Both cohorts were followed from the index date until the diagnosis of AD, death or 31 December 2013 (Fig. 1). The covariates included sex and age group (50–59, 60–69 and ≥70 years). According to the definition of urbanisation issued by the National Institutes of Health in Taiwan, all 365 townships in Taiwan are divided into 7 clusters according to the following variables: population density (people per square kilometre), proportion of the population with a college-level education or higher, proportion of individuals over 65 years old, number of agricultural workers, and number of physicians per 100,000 people. In the present study, townships of one or two clusters, three or four clusters, and five to seven clusters were categorised as levels 1, 2 and 3, respectively [34].

We also analysed AD-related co-morbidities, including hypertension (ICD-9-CM codes 401.1, 401.9, 402.10, 402.90, 404.10, 404.90, 405.1 and 405.9) [10], hyperlipidaemia (ICD-9-CM codes 272.0–272.9) [11], chronic kidney disease (ICD-9-CM codes 580, 581–589, 753, 403, 404, 250.4, 274.1, 440.1, 442.1, 447.3, 572.4, 642.1 and 646.2) [12], depression (ICD-9-CM code 311) [13], stroke (ICD-9-CM codes 433, 434 and 436) [14], diabetes mellitus (ICD-9-CM codes 250–250.3, 250.7) [22] and traumatic brain injury (ICD-9-CM codes 800–804, 850–854, 905.0, 950.1, 950.3, 907.0, 959.01, 959.9, 310.2 and V15.52) [15]. The Charlson comorbidity index (CCI), which contains 17 weighted co-morbidities, was also calculated for each participant [35].

### Statistical analysis

We used *t* tests and chi-square tests to compare the demographic and clinical characteristics of patients with CP with those of patients without CP. Univariate and multivariate stratified Cox regression models were then used to calculate HRs and 95% CIs. Multivariable models were adjusted for AD-related co-morbidities, CCI score, and urbanisation level. The Kaplan-Meier method was used to assess the AD survival probability between the exposed and unexposed cohorts. The log-rank test was used to compare differences between these two cohorts.

Sensitivity analysis was performed to identify patients diagnosed with CP ≥1 year following the diagnosis of AD, as well as the incidence of AD occurring ≥10 years following the diagnosis of CP [4]. To ensure the stability and accuracy of the statistical model, we excluded from the sensitivity analysis patients diagnosed with AD <1 and <10 years after the diagnosis of CP [36]. We used a mediation model to identify and explain the other pathways or processes underlying an observed relationship between CP (independent variable) and AD (dependent variable) via the hypothetical mediator of cerebrovascular disease (ICD-9 codes 430–438) [26]. All statistical analyses were performed using SAS version 9.3 (SAS Institute, Cary, NC, USA) and IBM SPSS Statistics version 22 (IBM, Armonk, NY, USA) software. The level of statistical significance was set at *p* < 0.05.

## Results

The baseline characteristics of the study sample are presented in Table 1. Patients with CP had a higher prevalence of hyperlipidaemia, depression and traumatic brain injury, as well as a CCI score and urbanisation level, than the unexposed cohort (all *p* < 0.01). The mean ages and follow-up times for the exposed and unexposed cohorts were 54.1 ± 10.5 vs. 54.2 ± 10.5 years and 11.9 ± 2.6 vs.12.2 ± 2.6 years, respectively.

**Table 1:** Demographic characteristics of the study cohort at baseline

Variable	Total		Chronic periodontitis				*p* value
			With		Without		
	*n*	%	*n*	%	*n*	%	
Total	27,963	100	9291	100	18,672	100	
Sex							
Female	13,119	46.92	4351	46.83	8768	46.96	0.8402
Male	14,844	53.08	4940	53.17	9904	53.04	
Age, years							
50–59	13,947	49.88	4638	49.92	9309	49.86	0.9705
60–69	8853	31.66	2945	31.70	5908	31.64	
≥70	5163	18.46	1708	18.38	3455	18.50	
Hypertension							
No	6654	23.80	2251	24.23	4403	23.58	0.2314
Yes	21,309	76.20	7040	75.77	14,269	76.42	
Hyperlipidaemia							
No	21,651	77.43	6935	74.64	14,716	78.81	<0.0001
Yes	6312	22.57	2356	25.36	3956	21.19	
Chronic kidney disease							
No	17,243	61.66	5697	61.32	11,546	61.84	0.4009
Yes	10,720	38.34	3594	38.68	7126	38.16	
Depression							
No	23,283	83.26	7619	82.00	15,664	83.89	<0.0001
Yes	4680	16.74	1672	18.00	3008	16.11	
Stroke							
No	21,316	76.23	7081	76.21	14,235	76.24	0.9652
Yes	6647	23.77	2210	23.79	4437	23.76	
Diabetes mellitus							
No	14,545	52.02	4627	49.8	9918	53.12	<0.0001
Yes	13,418	47.98	4664	50.2	8754	46.88	
Traumatic brain injury							
No	22,517	80.52	7562	81.39	14,955	80.09	0.0099
Yes	5446	19.48	1729	18.61	3717	19.91	
CCI score							
0	17,032	60.91	6423	69.13	10,609	56.82	<0.0001
1	7826	27.99	2206	23.74	5620	30.10	
2	3105	11.10	662	7.13	2443	13.08	
≥3	1685	6.03	499	5.37	1186	6.35	
Urbanisation level							
1	2896	10.36	890	9.58	2006	10.74	<0.0001
2	3675	13.14	1219	13.12	2456	13.15	
3	19,707	70.48	6683	71.93	13,024	69.75	

*CCI* Charlson comorbidity index

**Fig. 2:**
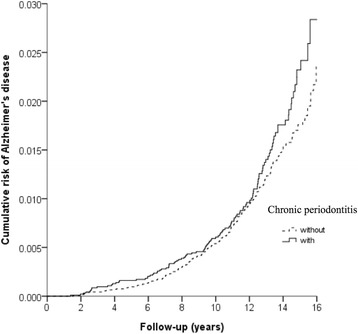
Kaplan-Meier model based on the Cox-regression analysis with the log-rank test for the cumulative risk of Alzheimer’s disease among the exposed and unexposed cohorts

Totals of 115 (1.24%) and 208 (1.11%) patients were diagnosed with AD in the exposed and unexposed cohorts, respectively (Fig. 1). Table 2 shows the Cox regression analysis of risk factors associated with the development of AD. Although patients with CP exhibited higher crude HRs (1.301, 95% CI 1.012–1.673, *p* = 0.0404) for the development of AD than those without CP, the adjusted HR was 1.297, indicating a lack of statistical significance (95% CI 0.995–1.692, *p* = 0.0547) (Table 2). Patients with depression, stroke and traumatic brain injury tended to have a higher risk for the development of AD (all *p* < 0.05). Moreover, the risk of AD was lower in patients with urbanisation level 2 and level 3 (both *p* < 0.05). Figure 2 depicts the Kaplan-Meier curve for the cumulative risk of AD in the exposed and unexposed groups. A significant difference was observed between the two groups once 10 years of CP exposure had been reached (*p* = 0.0264 by log-rank test).

**Table 2:** Covariates associated with Alzheimer’s disease at end of follow-up with univariate, multivariable and sensitivity analysis of Cox regression analysis

Variable	Univariate analysis			Multivariable analysis		
	Crude HR	95% CI	*p* value	Adjusted HR	95% CI	*p* value
1-year exclusion in a diagnosis of AD						
Chronic periodontitis						
Without	Reference			Reference		
With	1.301	1.012–1.673	0.0404	1.297	0.995–1.692	0.0547
10-year exclusion in a diagnosis of AD						
Chronic periodontitis						
Without	Reference			Reference		
With	1.364	1.079–1.725	0.0095	1.707	1.152–2.528	0.0077

*AD* Alzheimer’s disease

The multivariable analyses were adjusted for hypertension, hyperlipidaemia, chronic kidney disease, depression, stroke, traumatic brain injury, diabetes mellitus, Charlson comorbidity index score, urbanisation level

The results of the sensitivity analysis are presented in Table 2. We performed sensitivity analysis after excluding patients diagnosed with AD <1 year and <10 years after the diagnosis of CP. The association between CP and AD was significant after 10 years of CP exposure (adjusted HR 1.707, *p* = 0.0077). In statistics, a mediation model explains and underlies an observed relationship between an independent variable (IV) and dependent variable (DV) via the inclusion of a mediator (M). They have significance of a mediation model (IV → M, *p* < 0.001, 95% CI 3.292–3.763; M → DV, *p* = 0.0118, 95% CI 1.121–2.506; and IV → D, *p* = 0.0077, 95% CI 1.152–2.528). This result shows that cerebrovascular disease is a partial mediator between CP and AD. CP can also cause AD directly.

## Discussion

The present study is the first nationwide population-based matched-cohort study to demonstrate that patients with 10-year CP exposure exhibit an increased risk of developing AD (adjusted HR 1.707), regardless of co-morbidities, CCI score or urbanisation level. The prevalence of AD significantly increases with age, although AD in general is more common in women than in men [3]. AD is characterised by salient inflammatory features, microglial activation and increased levels of pro-inflammatory cytokines, which contribute to the inflammatory status of the central nervous system [37]. As a low-grade systemic disease, CP may involve the slow release of pro-inflammatory cytokines and CRP into the systemic circulation. This low-grade inflammation is thought to impact general systemic health and exacerbate other systemic disorders [38]. Therefore, CP may be a significant source of covert peripheral inflammation within the general population. Periodontitis has a tendency to infiltrate the systemic circulation with inflammatory mediators, thereby resulting in systemic disease. Researchers have proposed that periodontitis can lead to progression of AD by further increasing levels of pro-inflammatory cytokines and can lead to the invasion of micro-organisms from the dental plaque biofilm into the brain [28]. Moreover, these pro-inflammatory cytokines may penetrate the blood-brain barrier and induce neurodegenerative changes that ultimately influence the development of AD [26]. In the present study, we observed a significant correlation between CP and AD only after the 10-year follow-up for the initial diagnosis of CP. This finding supports the notion that pro-inflammatory factors due to CP may slowly and progressively induce neurodegenerative changes that lead to the development of AD. However, further study is required to verify this hypothesis.

In previous small and/or short-term cross-sectional studies, the authors examined only the associations between periodontal inflammation and AD [5, 27], without investigating the potential cause-and-effect relationship between the two. However, in a previous study, researchers reported that periodontitis is associated with an increased risk of developing dementia [33]. Moreover, a 10-year clinical observational study conducted by Tzeng et al. revealed that patients with at least 8 years of periodontal problems exhibited a significantly higher risk of developing dementia and neurodegenerative diseases than healthy unexposed groups [4]. Despite these crucial findings, neither of the aforementioned studies identified the role of periodontitis in AD development. The present population-based study is the first to demonstrate a significantly increased risk of AD after 10-year CP exposure. Our findings support the notion that infectious diseases associated with low-grade inflammation, such as CP, may play a substantial role in the pathogenesis of AD [39]. In mediation analysis, the role of cerebrovascular disease is as a partial mediator, and CP can also cause AD directly. Systemic inflammation caused by periodontal pathogens, such as *Porphyromonas gingivalis* and *Streptococcus sanguinis*, may be a factor in endothelial dysfunction and vascular disease [26]. The mediator model facilitates a better understanding of the relationship between CP and AD. However, it needs more relevant and further investigation to confirm and clarify the pathway.

Co-morbidities including depression, stroke and traumatic brain injury were directly associated with the risk of developing AD in the present study, in accordance with the findings of previous studies. Burke et al. reported that depression was associated with AD in a group of participants who were initially cognitively asymptomatic [13], whereas additional studies have revealed a significant association between stroke/brain damage and dementia [14, 15]. Our findings also indicate that urbanisation level 1 was a significant risk factor for AD. This finding may be explained by differences between urban and rural lifestyles, availability of medical resources and convenience of medical access [3, 40]. Patients with AD also exhibit impairments in chewing function due to progressive neurodegeneration, increasing the risk of periodontal problems [41, 42]. Hence, proper dental care and oral rehabilitation are necessary to improve masticatory function in this patient population.

The present study possesses several advantages over previous work. Firstly, we used a nationwide database, which allowed us to examine a large sample of patients over a 16-year follow-up period. Secondly, because the Taiwanese NHIRD provides continued coverage for the whole population of Taiwan and randomises recruits, in the present study, we were able to minimise the influence of bias associated with data collection, region and institution. Thirdly, the use of the NHIRD eliminated the need to minimise patients in the cohort who were lost to follow-up and enabled us to obtain large, geographically dispersed samples of patients with varying socio-demographic characteristics [34]. Lastly, we defined AD using strict criteria (ICD-9-CM code 331.0) to ensure the accuracy of our statistical analysis.

However, the present study also possesses some limitations of note. Firstly, we could not clarify the medical records of all missing recruited CP and AD subjects, because all the medical records from the NHIRD were de-identified due to ethical considerations. It is possible that the incidence of CP or AD was underestimated in our study because patients who had not received medical records during the study period were excluded. Although this would result in fewer patients with less severe forms of CP or AD in our sample, the exposed-unexposed matched design would also diminish this bias. Secondly, the nationwide database used in the present study did not allow us to obtain data regarding the severity of AD. Furthermore, AD and dementia may overlap in this study because we were not able to clarify the medical records of all defined AD, owing to all the medical records from the NHIRD being de-identified due to ethical considerations. Thirdly, the broad age categories are important variable indexes. However, because of this limitation, there may be residual confounding by age [4]. Finally, in detailed demography concerning smoking habits, the NHIRD did not provide personal information regarding certain and definite variables relevant to our research [43]. Moreover, although education is an important variable index of AD, the education level of individuals in the NHIRD is not available. Further research is needed to explain the complex relationship between urbanisation and education.

## Conclusions

Our findings demonstrate that 10-year CP exposure was associated with a 1.707-fold increase in the risk of developing AD. These findings highlight the need to prevent progression of periodontal disease and promote healthcare services at the national level.

## Abbreviations

AD: Alzheimer’s disease
CCI: Charlson co-morbidity index
CP: Chronic periodontitis
CRP: C-reactive protein
ICD-9-CM: International Classification of Diseases, Ninth Revision, Clinical Modification
IRB: Institutional review board
NHIRD: National Health Insurance Research Database
